# Segmental Scleral Buckle: A Novel Strategy for Addressing Early Recurrent Inferior Retinal Detachment in Silicone Oil-Filled Eyes

**DOI:** 10.3390/life15030475

**Published:** 2025-03-16

**Authors:** Luca Ventre, Antonio Valastro, Erik Mus, Fabio Maradei, Giulia Pintore, Gabriella De Salvo

**Affiliations:** 1 Ophthalmology Department, Beauregard Hospital, Azienda USL della Valle d’Aosta, Via L. Vaccari 5, 11100 Aosta, Italy; 2 Southampton Eye Unit, University Hospital Southampton Foundation Trust, Southampton SO16 6YD, UK; 3 University of Southampton, Southampton SO16 6YD, UK

**Keywords:** pars plana vitrectomy, recurrent retinal detachment, retinal detachment, segmental scleral buckle, silicone oil tamponade

## Abstract

Recurrence of retinal detachment (RD) following pars plana vitrectomy (PPV) with silicone oil tamponade is a surgical challenge. This study proposes a novel approach utilizing segmental scleral buckle to manage early recurrences, especially in inferior quadrants. A retrospective case series of four patients with early recurrent inferior RD post-PPV with silicone oil tamponade was conducted. The segmental scleral buckle technique, with or without subretinal fluid drainage, was employed. Clinical and surgical data were collected, including visual outcomes and complications. No intraoperative or postoperative complications were observed during the 6-month follow-up period. Visual acuity remained stable, and retinal reattachment was achieved in 100% of cases after silicone oil removal. Segmental scleral buckle emerges as a promising technique for managing early recurrent inferior RD in silicone oil-filled eyes. The technique demonstrates favorable outcomes, including retinal reattachment and visual acuity stability, without significant complications. Further studies are warranted to validate its efficacy and establish standardized protocols.

## 1. Introduction

Rhegmatogenous retinal detachment (RRD) is one of the leading causes of vision loss, with an incidence of approximately 1 in 10,000 [1,2].

RRD occurs when a break in the retina, often caused by posterior vitreous detachment or trauma, allows liquefied vitreous to pass into the subretinal space, leading to detachment of the neurosensory retina from the retinal pigment epithelium [3].

Treatment of rhegmatogenous retinal detachment involves identifying and closing all retinal breaks using techniques such as laser retinopexy, pneumatic retinopexy, scleral buckling, or pars plana vitrectomy (PPV) [4].

Pars plana vitrectomy involves the removal of the vitreous body to eliminate all tractional forces on the retina, followed by flattening of the detached retina and treatment of all identified retinal breaks with laser photocoagulation or cryotherapy. At the end of the procedure, a tamponade agent—air, gas, or silicone oil (SiO)—is introduced into the vitreous cavity to stabilize the retina and promote reattachment [4].

Recurrence of retinal detachment (RD) may develop at different postoperative times following PPV for RRD. When the retinal detachment recurrences occur within the first 6 weeks post-primary PPV, we refer to those as “early recurrences”; when they occur after the first 6 weeks from primary surgery, they are referred to as “late recurrences” [5,6].

In this study, we focus specifically on early recurrences of retinal detachment in eyes filled with silicone oil tamponade.

Several factors contribute to early re-detachment in SiO–filled eyes, including underfilling the vitreous chamber with silicone oil, incomplete release of vitreoretinal tractions at the edge of the break, the buoyancy characteristics of silicone oil itself, and the development of early (immature) proliferative vitreoretinopathy. Inadequate SiO filling can result in a meniscus of residual fluid accumulating inferiorly, reducing the tamponade effect on the lower retina [6]. Additionally, residual vitreoretinal tractions at the edge of the break may lead to detachment in the lower quadrants of the retina, particularly when the silicone oil tamponade is insufficient [6].

Conventional surgical interventions to address these recurrences involve a re-do vitrectomy (revitrectomy) or a 360-degree scleral buckle [6,7].

Revitrectomy typically involves the removal of silicone oil, revision of the vitrectomy to release any remaining vitreoretinal tractions, reflattening of the retina using air or perfluorocarbon liquids, and possibly revisiting the endolaser performed during the previous surgery, either to reinforce treatment of pre-existing breaks or to address any new breaks that may have formed or been missed during primary surgery. In most cases, silicone oil is then added [6].

The 360-degree scleral buckle procedure involves performing a 360-degree peritomy, isolating the four rectus muscles (superior, medial, lateral, and inferior), placing a silicone band around the globe (beneath the previously isolated rectus muscles), optionally performing a transscleral drainage puncture to evacuate subretinal fluid, tightening the band to achieve the desired indentation, and applying cryotherapy (or, alternatively, laser treatment in the postoperative period) to retinal breaks. The procedure is completed by closing the conjunctiva [8].

However, scleral buckle has drawbacks, such as prolonged surgical time, increased refractive error, and a higher risk of complications like diplopia and anterior segment ischemia [9]. On the other hand, revitrectomy carries the risk of repeating previous errors, such as inadequate filling of silicone oil or failure to fully address residual tractional forces from remaining membranes, which may compromise the success of the procedure.

Current guidelines for managing recurrent RD in silicone oil-filled eyes are limited [6], and the choice of surgical approach, and associated outcomes have not been thoroughly defined.

Thus, the aim of this study is to present an alternative surgical approach for treating early inferior retinal detachment in silicone oil-filled eyes, avoiding the need for further pars plana vitrectomy and/or a 360-scleral buckle. We propose the use of an inferior, segmental (circumferential) scleral buckle, with or without external drainage of subretinal fluid (depending on the amount of subretinal fluid present). In cases of shallow or “flat” detachments, we opted not to drain the subretinal fluid and instead placed the buckle alone to avoid potential iatrogenic retinal damage from the drainage puncture. However, in cases with a significant amount of subretinal fluid, drainage was performed, as the risk of iatrogenic damage from the puncture was lower, to enhance the inferior tamponade effect. By applying pressure to the eye wall, the sponge pushes the retina into contact with the silicone oil but also helps to counteract tractional forces. At the same time, the silicone oil provides a counterpressure against the retina, effectively stabilizing its position. This dual action also eliminates the meniscus of residual fluid that may persist due to accidental underfilling, thereby enhancing the overall success of the procedure.

## 2. Materials and Methods

This retrospective analysis of a series of cases was conducted at Beauregard Hospital, Aosta between November 2021 and July 2023. All surgeries were performed by an experienced vitreoretinal surgeon (L.V.). The study was conducted in adherence to Italian bioethical guidelines and the Declaration of Helsinki. All patients received detailed information and were provided with informed consent regarding the type of surgical procedure performed.

To be enrolled in the study, the four consecutive patients had to meet the following eligibility criteria: (1) recurrent inferior retinal detachment occurring within one month after primary surgery or “early recurrence” (inferior RD defines a RD localized in the quadrants comprising the inferior six clock hours of the retinal clock—3 o’clock to 9 o’clock); (2) silicone oil used as tamponade in primary surgery; (3) recurrence of inferior RD did not involve the macular region (early inferior recurrence with macula-on RD). Exclusion criteria included the following: (1) relaxing retinectomies during the initial surgery; (2) scleral buckling procedure during the primary vitreoretinal surgery; (3) recurrence of macula-off RD.

All patients underwent a thorough ocular assessment, including best-corrected visual acuity (BCVA) assessment, anterior segment examination, intraocular pressure (IOP) measurement, fundus examination, fundus photography (Cobra+, CSO, Florence, Italy), and spectral-domain optical coherence tomography (SD-OCT) (Spectralis Heidelberg Engineering, Heidelberg, Germany). These examinations were performed before the primary surgery, within one month of primary surgery, and then at one week, one month, three months, and six months after segmental scleral buckle surgery.

Further data regarding age, gender, pre-reoperation BCVA, timeframe between primary PPV and reoperation, extent of retinal detachment, recurrence factors, location of retinal breaks, surgical procedures undertaken, retinal reattachment rate, final visual outcome, postoperative intraocular pressure (IOP), and intra- and postoperative complications were collected.

The surgical technique used in all four patients was inferior segmental scleral buckle with (or without) drainage of subretinal fluid; the latter was performed in two out of four patients.

The steps of the surgery are described in detail below:

### 2.1. STEP 1–Identification and Isolation of the Inferior, Medial, and Lateral Rectus Muscles

**Figure 1:**
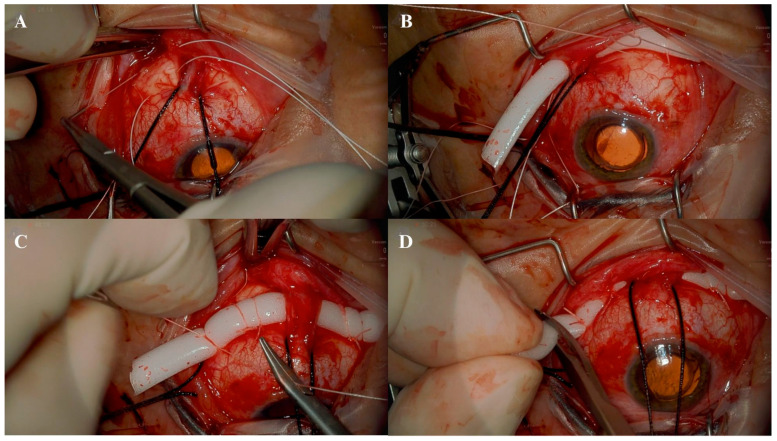
Intraoperative view of the surgical steps of an inferior segmental scleral buckle. (**A**): Placement of 5/0 Tycron scleral horizontal mattress sutures in the inferotemporal quadrant; (**B**): positioning of the 5 mm × 3 mm silicone sponge beneath the rectus muscles and under the previously placed horizontal mattress sutures; (**C**): tightening of the four horizontal mattress sutures to achieve the desired indentation; (**D**): trimming of the excess portion of the silicone sponge.

The procedure begins with a 180-degree lower conjunctival peritomy to gain access to the underlying scleral area. The conjunctiva and Tenon’s capsule are carefully dissected using Westcott scissors, isolating the sub-Tenon space and exposing the sclera beneath. Following this, the inferior, medial, and lateral rectus muscles are identified and isolated individually using a fenestrated muscle hook. This step facilitates the placement of a reverse-mounted suture (2/0 silk suture) around the belly of each muscle to secure and maneuver them as needed during the subsequent stages of surgery. This careful preparation ensures optimal exposure and access to the surgical site (Figure 1A).

### 2.2. STEP 2–Placement of Horizontal Mattress Sutures

Once the surgical field has been prepared, scleral marking is performed at a distance of 12 mm from the limbus to guide the correct placement of sutures. Two horizontal mattress sutures are then placed in each lower quadrant using a 5/0 Tycron suture (Figure 1B). These sutures serve as the primary anchors for securing the scleral buckle and ensuring proper indentation. Accurate placement of these sutures is critical for achieving the desired tamponade effect in the affected retinal areas.

### 2.3. STEP 3–Positioning of the Scleral Buckle

The scleral buckle, consisting of a 5 mm × 3 mm sponge, is positioned beneath the horizontal mattress sutures that were previously placed in the inferior retinal quadrants, typically spanning the 3 to 6 o’clock positions (Figure 1C). If subretinal fluid drainage is performed, it is performed at this stage to facilitate retinal reattachment.

The horizontal mattress sutures are then tightened properly to induce the desired level of indentation in the inferior retinal quadrants (Figure 1C). Excess sponge material is carefully cut to avoid unnecessary bulk or interference with the surrounding ocular structures (Figure 1D). Finally, the conjunctiva is sutured.

## 3. Results

This series includes four pseudophakic eyes of four patients (three females and one male) with early inferior recurrent retinal detachment, three of whom initially presented with primary macula-off rhegmatogenous retinal detachment, while in one case, the macula remained attached during the primary retinal detachment (macula-on) (Table 1).

Primary surgery consisted of PPV with silicone oil as tamponade in all four patients: Patient 1 was an 80-year-old male who presented with a macula-on (Stage 2 according to the OCT-based classification of Melo et al. [10]) retinal detachment accompanied by a giant retinal tear located in the superior quadrants, extending from 10 o’clock to 2 o’clock. Patient 2 was a 63-year-old female who presented with a longstanding subtotal retinal detachment (Stage 4 according to the OCT-based classification of Melo et al. [10]), characterized by advanced proliferative vitreoretinopathy (PVR) of grade C; two star folds—one at 5 o’clock and one at 7 o’clock—located posterior to the equator were present. According to the Machemer classification [11], the proliferative vitreoretinopathy was graded as PVR C3; these star folds were peeled using a monomanual technique employing ILM forceps without the use of staining; the detachment was associated with multiple retinal breaks extending circumferentially around the retina, spanning a full 360 degrees. Patient 3 was a female with a longstanding macula-off retinal detachment (Stage 3A according to the OCT-based classification of Melo et al. [10]); the detachment was associated with a retinal horseshoe tear located at 5 o’clock, which was observed in the context of lattice degeneration. Patient 4 was also a female who presented with a longstanding macula-off retinal detachment (Stage 3A according to the OCT-based classification of Melo et al. [10]); in her case, the detachment was linked to a retinal horseshoe tear at 11 o’clock, similarly occurring in an area of lattice degeneration. During primary PPV, the surgeon confirmed the presence of the posterior hyaloid by injecting triamcinolone acetonide (0.1–0.3 mL at a concentration of 40 mg/mL). In all four cases, a PVD was already present. In the last two patients, the decision to use silicone oil as a tamponade was influenced by the high altitudes of their place of residence, with both living at elevations exceeding 2000 m above sea level. At such altitudes, the use of gas tamponade would have been contraindicated due to the significant risk of gas expansion caused by reduced atmospheric pressure, which could lead to postoperative complications, including elevated intraocular pressure (ocular hypertension).

In all four cases, varying degrees of hypofilling of the vitreous cavity by silicone oil were observed. In Patient 2, previously undetected (or newly formed) microholes were identified in the inferior quadrants, which likely contributed to the early recurrence of the detachment. In Patient 3, early (immature) PVR was noted, along with inferior microholes; in combination with the incomplete filling of the vitreous cavity by silicone oil, these factors likely led to an early inferior re-detachment.

Neither intraoperative nor postoperative complications were observed during the follow-up examinations at 1 week, 1 month, 3 months, and 6 months after the segmented scleral buckle procedure (Table 1). As indicated in Table 1, visual acuity exhibited stability throughout the 6-month follow-up period. Axial length remained unchanged after segmental scleral buckle. Furthermore, the reattachment rate achieved was 100% during the 6-month follow-up period after the removal of silicone oil, a procedure conducted within 3 months of the primary surgery.

## 4. Discussion

The success rate in addressing complex rhegmatogenous retinal detachment (RRD) has significantly improved due to the adoption of advanced pars plana vitrectomy (PPV) techniques and the application of silicone oils as tamponade. These advancements have provided better retinal stabilization and facilitated the management of even the most challenging cases. However, despite these technical improvements, the recurrence rate of retinal detachment can reach up to 10% of cases [12].

Recurrences of retinal detachment can occur at different times following primary surgery and are typically categorized as either “early recurrences”, which occur within the first 6 weeks, or “late recurrences”, which manifest more than 6 weeks after the initial procedure [5,13]. Understanding the distinction between these types of recurrences is essential as the underlying causes and management strategies differ significantly.

Early recurrences usually impact the lower retinal quadrants, and several factors contribute to their occurrence, many of which are related to technical aspects of the primary surgery. One common issue is inadequate filling of the vitreous cavity with silicone oil during the initial surgery. Silicone oil, being lighter than water, rises to the superior part of the vitreous cavity when the patient is in an upright position. This buoyancy provides an effective tamponade for the upper retinal quadrants but leaves the inferior sectors less protected. As a result, recurrences of retinal detachment in eyes with silicone oil tamponade often occur in the inferior retinal sectors.

This issue is further exacerbated when the vitreous cavity is underfilled with silicone oil, leading to the formation of a meniscus of water in the inferior quadrants [14]. The rounded shape of the silicone oil bubble compounds the problem as part of its volume is utilized to form this meniscus rather than maintaining direct contact with the retina [15]. This lack of tamponade efficiency, particularly in the lower quadrants, increases the likelihood of recurrent detachment [16]. Another significant factor is the presence of undetected retinal breaks or microholes during the primary surgery. These lesions may go unnoticed during primary surgery and remain untreated. Due to the buoyant nature of silicone oil, these breaks are less effectively tamponaded when localized in the inferior retina, allowing SRF to seep through and contributing to the recurrence of RD.

Additionally, early recurrences may result from an incomplete release of vitreoretinal tractions at the edge of breaks during the primary surgery. Residual tractions can exert pulling forces on the retinal surface, particularly in the inferior quadrants where the tamponade effect of silicone oil is less. This mechanical stress can lead to new tears or exacerbate existing ones, resulting in recurrent detachment.

Another possible cause of early re-detachment could be the development of early (immature) proliferative vitreoretinopathy.

The combination of these factors—insufficient silicone oil filling, undetected retinal breaks, unrelieved vitreoretinal tractions, and immature PVR—can act independently or synergistically to cause early recurrences. Each of these issues is particularly problematic in the inferior retinal quadrants, where silicone oil provides less reliable support.

On the contrary, late recurrences of retinal detachment are primarily attributed to the development of mature PVR, which is a complex process characterized by the proliferation, migration, and contraction of retinal pigment epithelial (RPE) cells, inflammatory cells, and fibroblasts [17]. This pathological response can lead to the formation of fibrotic membranes that exert traction on the retina, causing the development of new retinal breaks or reopening previously treated ones. Additionally, the contraction of these membranes often results in a progressive shortening and distortion of the retinal tissue, further complicating the anatomical and functional outcomes of retinal reattachment procedures [18].

The surgical approach to managing recurrent RD in silicone oil-filled eyes necessitates a comprehensive evaluation of the underlying causes of the initial surgical failure. Surgeons must carefully assess the extent and location of PVR, residual tractional forces, undetected or new retinal breaks, and the effectiveness of the tamponade. In certain cases, reoperations can be performed without removing the silicone oil, focusing on peeling PVR membranes and reinforcing the retinal attachment using endolaser or cryotherapy [19]. However, in more complex cases, the procedure involves the removal of the silicone oil to allow better access to the retina for a more thorough intervention. This often includes performing extensive PVR peeling to relieve traction, reflattening the retina using perfluorocarbon liquids, addressing any new or preexisting retinal breaks, and ensuring adequate tamponade. Depending on the severity and location of the detachment, tamponading agents such as silicone oil (for long-term support) or gas (for temporary but effective tamponade) are used to stabilize the retina [20].

The success of these reoperations relies heavily on meticulous surgical planning, careful intraoperative handling of delicate retinal tissue, and the ability to adapt the surgical approach based on the extent of PVR and other contributing factors to the recurrent detachment.

A different surgical approach in cases of RD recurrence is external. In this case, scleral buckling (SB) enhances the tamponade effect of silicone oil (SiO) on the inferior retina by establishing contact between the peripheral retina and the silicone oil bubble, reducing traction on the retina circumferentially and providing support to the retinal breaks [16].

Segmental scleral buckle represents a straightforward, time-efficient, and cost-effective surgical technique that, despite its simplicity, proves to be highly effective in managing and addressing retinal detachments [21].

Typically utilized for the treatment of primary rhegmatogenous retinal detachments involving a single retinal break, this technique offers localized support specifically at the site of the break [21]. By focusing on the affected area, it eliminates the need for a more extensive 360-degree buckle procedure, reducing surgical time and minimizing potential complications while still ensuring effective retinal reattachment and stabilization [22].

In early recurrent inferior retinal detachments, as previously mentioned, detachment is caused by failure of the retinal tamponade provided by the silicone oil bubble in the inferior quadrants and/or by residual, not completely relieved vitreoretinal tractions, which may or may not have created new rhegmatogenous lesions in the inferior retinal sectors. These new lesions, not being adequately tamponaded—potentially due to underfilling of the eye with silicone oil—can lead to a recurrence of inferior retinal detachment. Therefore, placing a segmental buckle in the lower 180 degrees of the retina would create an indentation in the inferior quadrants, bringing the retina closer to the silicone oil bubble and thereby enhancing the tamponade effect of the silicone oil itself. Additionally, this approach helps relieve any residual vitreoretinal tractions or address inferior retinal breaks, further contributing to the stabilization and reattachment of the retina.

The advantages of this technique over a 360-degree scleral buckle and additional vitrectomy are numerous, including minimal alterations in refractive error, reduced surgical time—typically requiring no more than 60 min for completion—lower invasiveness compared to performing a revitrectomy, decreased overall cost of the procedure, a significantly reduced risk of postoperative diplopia as the surgery involves only the inferior rectus muscle, and a relatively low risk of both anterior and posterior segment ischemia, making it a safer and more efficient option for managing early recurrent inferior retinal detachments.

As the study is retrospective in nature, includes a case series of only four eyes, and has a relatively short follow up of six months, further standardized, randomized, and multicentric clinical trials are needed to validate our findings. Nevertheless, we found the use of segmental scleral buckle in the early recurrence inferior RD encouraging, not only due to its positive anatomic and refractive outcomes but also in most efficiently managing precious theatre time with minimal postoperative patient recovery time.

## 5. Conclusions

In conclusion, this case series highlights the efficacy of segmental scleral buckle as a novel and promising strategy for addressing early recurrent inferior retinal detachment in silicone oil-filled eyes. The technique demonstrated favorable outcomes, including retinal reattachment and stability in visual acuity, without significant complications during the follow-up period. The advantages of segmental scleral buckle over traditional 360-degree scleral buckling and repeated pars plana vitrectomy include reduced surgical time, lower risk of refractive errors and postoperative complications, and overall cost-effectiveness. However, further large-scale studies are necessary to validate these findings and establish standardized protocols for wider clinical application.

## Figures and Tables

**Table 1:** Clinical and surgical data for patients with early recurrent inferior retinal detachment. Summary of retinal detachment characteristics and postoperative outcomes. RD = Retinal Detachment; VTC = Vitrectomy; sSB = segmental Scleral Buckle; SiO = Silicone Oil.

	OCT-Based Stage of Primary RD (Melo et al. [10])	Intraop./Postop. Complications	BCVA Pre-VTC	BCVA 1-Week Post-VTC	BCVA 1-Week Post-sSB	BCVA 6-Months Post-SiO Removal
**Patient 1**	2	No	20/30	20/25	20/25	20/20
**Patient 2**	4	No	HM	20/70	20/70	20/40
**Patient 3**	3A	No	CF 1 m	20/50	20/50	20/30
**Patient 4**	3A	No	CF 3 m	20/40	20/40	20/25

## Data Availability

The data presented in this study are available on request from the corresponding author. The data are not publicly available due to privacy concerns.
